# Kidney Transplant Recipient Behavior During the Early COVID-19 Pandemic: A National Survey Study in Norway

**DOI:** 10.1016/j.xkme.2021.09.006

**Published:** 2021-11-17

**Authors:** Kjersti B. Blom, Anders Åsberg, Ivar Sjaastad, Karl T. Kalleberg, Arne Søraas, Karsten Midtvedt, Jon A. Birkeland

**Affiliations:** 1 Department of Nephrology, Oslo University Hospital, Ullevål, Oslo, Norway; 2 Institute for Experimental Medical Research, Oslo University Hospital, Ullevål, Oslo, Norway; 3 Center for Heart Failure Research, Oslo University Hospital, Ullevål, Oslo, Norway; 4 Department of Transplantation Medicine, Oslo University Hospital, Ullevål, Oslo, Norway; 5 Department of Cardiology, Oslo University Hospital, Ullevål, Oslo, Norway; 6 University of Oslo, Oslo, Norway; 7 KG Jebsen Center for Cardiac Research, University of Oslo, Oslo, Norway; 8 Age Labs AS, Oslo, Norway; 9 Department of Microbiology, Oslo University Hospital, Case Comprehensive Cancer Center, Oslo, Norway

**Keywords:** COVID-19, everyday life behavior, immunosuppression, infectious disease, kidney transplant recipient

## Abstract

**Rationale & Objective:**

Studies published from countries with a high prevalence of COVID-19 have found increased incidence and a more severe disease course of coronavirus disease 2019 (COVID-19) in kidney transplant recipients than in the general population. We investigated how the first wave of the COVID-19 pandemic affected the everyday life of kidney transplant recipients in a country with a low infection burden.

**Study Design:**

Prospective case-control study.

**Setting & Participants:**

All adult kidney transplant recipients in Norway with a functioning graft and listed in the public phone registry (n = 3,060) and a group of randomly recruited individuals >18 years from the general population (n = 20,000) were invited to participate in the study by an SMS text message. In parallel, all kidney transplant recipients in Norway were invited to measure severe acute respiratory syndrome coronavirus 2 (SARS-CoV-2) IgG from mid-June to October 2020.

**Predictors:**

The participants were asked to fill out a questionnaire focused on everyday life, travel history, exposure to known COVID-19 cases, and demographics.

**Analytical Approach:**

Groups were compared with independent tests using 2-sided 0.05 significance levels.

**Results:**

A total of 1,007 kidney transplant recipients and 4,409 controls answered the questionnaire. The kidney transplant recipients reported being more concerned about SARS-CoV-2 infection (27%) than the control group (7%; *P* value < 0.001); ie, they behaved more carefully in their everyday life (not going to the grocery store, 5.9% vs 0.9%, *P* < 0.001; keeping at least 1 meter distance, 16.6% vs 5.8%, *P* < 0.001). Of the kidney transplant responders, 81% had a SARS-CoV-2 IgG taken; all were negative.

**Limitations:**

Mortality data is not reliable because of the low number of SARS-CoV-2 infected kidney transplant recipients in Norway. The relatively low questionnaire response rate for kidney transplant recipients is not optimal.

**Conclusions:**

The questionnaire shows that kidney transplant recipients have behaved more carefully compared with the general population with less social interaction and a very high degree of adherence to governmental advice.

## Graphical abstract

**Figure:**
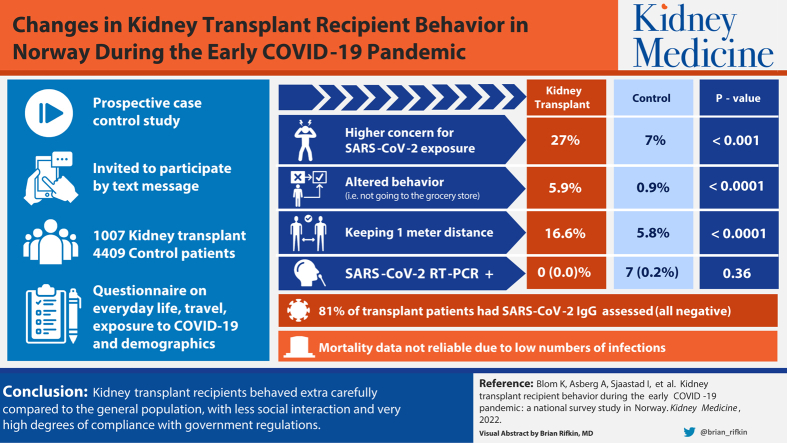

**Plain-Language Summary**

Studies from countries with a high incidence of COVID-19 disease have shown increased incidence and a more severe disease course of COVID-19 in kidney transplant recipients than in the general population. We investigated how the COVID-19 pandemic has affected the Norwegian kidney transplant recipient population by inviting all Norwegian kidney transplant recipients and a control group to answer an SMS questionnaire focused on everyday life behavior. In parallel, all kidney transplant recipients in Norway were invited to measure SARS-CoV-2 IgG. We found that kidney transplant recipients have behaved more carefully compared with the general population. Also, kidney transplant recipients have not had an increased incidence of COVID-19 compared with the general population, in contrast to previous findings in countries with a high prevalence of COVID-19.

Coronavirus disease 2019 (COVID-19) rapidly spread worldwide and was declared a pandemic by the World Health Organization in March 2020.^1^ It was soon recognized that healthy individuals of any age could experience severe illness and death. This led to different scenarios varying from country to country but often involving office and factory shut-downs and restrictions placed on social interactions. The media was flooded daily with COVID-19 horror stories and scenarios. People were scared, and medical knowledge was limited. As more COVID-19 specific information was encountered, most of the patients admitted to intensive care units were found to be older adults, often with underlying medical conditions.^2^, ^3^, ^4^, ^5^, ^6^ Comorbid conditions such as hypertension, diabetes, cardiovascular disease, and chronic kidney disease were acknowledged as risk factors for severe COVID-19. These risk factors are frequently found in kidney transplant recipients. The need for life-long immunosuppression is a challenge for fighting viral infections and may pose a specific risk for a more severe course of COVID-19 in kidney transplant recipients. Additionally, frequent hospital medical surveillance check-ups are needed, leading to regular contact with the health care system, which might increase the risk of infection during an ongoing pandemic. Recently, studies published from countries with a high prevalence of COVID-19 support this theory, as they found increased incidence and a more severe disease course of COVID-19 in kidney transplant recipients than in the general population.^7^, ^8^, ^9^ The mortality rate of kidney transplant recipients infected with severe acute respiratory syndrome coronavirus 2 (SARS-CoV-2) is approximately 20%, among the highest reported for any population.^10^ However, more studies are needed on the disease course of COVID-19 in kidney transplant recipients, especially in countries with a low prevalence of COVID-19. As consulting physicians in a country with a low infection burden, we wanted to investigate the impact of the COVID-19 pandemic on kidney transplant recipients’ everyday lives. We used a questionnaire and compared possible behavioral changes in the kidney transplant recipient population with an age-matched population. In addition, we performed a national screening of the incidence of asymptomatic SARS-CoV-2 infection in the kidney transplant recipient population during the first wave of COVID-19 by SARS-CoV-2 IgG measurements from mid-June to October 2020.

## Methods

### Study Population and Area

**Figure 1:**
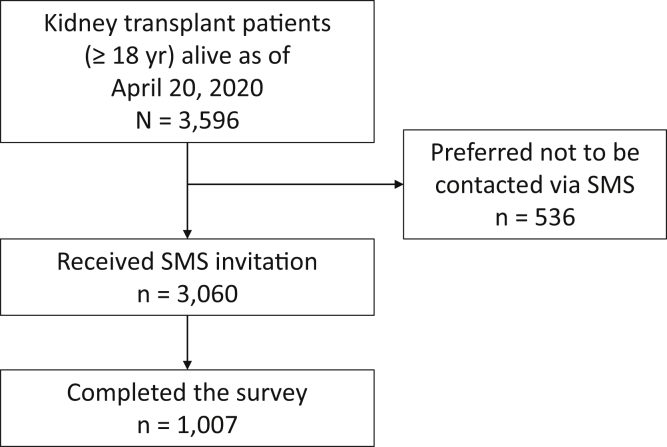
Overview of the inclusion of kidney transplant recipients.

All adult kidney transplant recipients (n = 3,596) with a functioning graft as of April 20, 2020, and a signed informed consent stating that data in the Norwegian Renal Registry (NRR) may be used for research were considered eligible to participate in the study. A search in the public phone registry among the 3,596 recipients yielded 3,060 to be eligible since 536 had reservations toward phone contact. Thus, 3,060 recipients were invited to participate in the study by SMS text messages. The questionnaire was available by logging on to a secure digital platform through a smartphone or a computer (Fig 1). A group of randomly recruited individuals >18 years (n = 20,000) from the general Norwegian population received the same SMS.

On March 13, 2020, the Norwegian authorities instituted a national lockdown, which entailed the closure of schools, preschools, restaurants, entertainment, and public gatherings. The authorities retracted entry visas and dismissed foreigners at the borders. The SMS invitation to the first questionnaire was sent out on April 24, followed by up to 4 reminders (answers received after May 27 were omitted). To those responding to the first SMS invitation, a second questionnaire was sent out on July 3, followed by up to 3 reminders (answers received after August 12 were omitted).

The questionnaire focused on behavior in everyday life, travel history, exposure to known COVID-19 cases, living arrangements, history of diseases, and self-reported test results (Table 1). The first questionnaire focused on the participants’ behavior in the 2 weeks before the lockdown or, if applicable, before they felt ill or were SARS-CoV-2 nasopharynx swab tested. The participants were also asked if they had experienced any symptoms of “light infections” the past 6 months (ie, upper respiratory tract infections not requiring hospitalization, cystitis, or infection not requiring antibiotics). The follow-up questionnaire focused on the period between July 7 and August 12.

**Table 1:** Demographic Data for Adult (≥18 y) Norwegian Kidney Transplant Recipients Who Answered the Questionnaire by April 20, 2020, (Kidney Transplant Responders) and the Control Group

	Kidney Transplant Responders (n = 1,007)	Control (n = 4,409)	*P* Value
Age (y)	56.6 ± 12.6	51.0 ±15.3	<0.001
Missing data	0	0	
BMI (kg/m^2^)	26.6 ± 5.6	26.2 ± 4.5	>0.99
Missing data	44	1,356	
Age over 65 y	274 (27.2%)	955 (21.6%)	<0.001
Missing data	0	0	
BP ≥140/90 mm Hg and/or antihypertensive drugs	864 (88.7%)	406 (14.2%)	<0.001
Missing data	34	1,548	
SARS-CoV-2 PCR positive	0 (0.0%)	7 (0.2%)	0.36
Missing data	0	0	
Residents of “red areas”^a^	692 (71.1%)	3,457 (79.7%)	<0.001
Missing data	34	69	
Income			
Missing data	238	1,193	
<$35,000	59 (7.7%)	150 (4.7%)	0.002
$35,000-$70,000	225 (29.3%)	757 (23.5%)	0.001
$70,000-$116,000	265 (34.5%)	1,120 (34.8%)	0.83
>$116,000	220 (28.6%)	1,189 (37.0%)	<0.001
Education level			
Missing data	439	2,698	
Primary and/or lower secondary school: 7-10 years total	31 (5.5%)	60 (3.5%)	0.05
Upper secondary school: 12 years total	225 (39.6%)	535 (31.3%)	<0.001
Higher education: less than 4 years	151 (26.6%)	503 (29.4%)	0.22
Higher education: 4 years or more	161 (28.3%)	613 (35.8%)	0.001

*Note:* Data are presented as mean (± standard deviation) or numbers (%). Data analyzed with R and with independent samples *t* test or χ^2^ test. Abbreviations: BP, blood pressure; BMI, body mass index; PCR, polymerase chain reaction.

a “Red areas”: geographical areas with ≥40 contaminated/100,000 inhabitants per ±2 weeks from March 13, 2020.

Simultaneous to the SMS study, all adult kidney transplant recipients (n = 3,596) with a functioning graft and signed informed consent for registration in NRR were invited (by personal letter) to have a SARS-CoV-2 IgG blood test performed (free of charge) during mid-June to October 2020. The test was most often performed in conjunction with a regular kidney transplant surveillance check-up. SARS-CoV-2 IgG measurements were initially performed by a multiplexed bead-based flow cytometric assay as previously described.^11^ The test had 100% specificity and 84% sensitivity. Positive and uncertain results were double-checked with a Roche Elecsys Anti-SARS-CoV-2 assay.^12^

Demographic data on kidney transplant recipients who answered the questionnaire (kidney transplant responders) were compared with all remaining adult kidney transplant recipients registered in NRR to evaluate whether the kidney transplant responders were representative of the whole group (Table 2). Since the virus spread differently in urban versus rural areas, a comparison was also made concerning the geographic distribution within areas with a high or low incidence of COVID-19. This comparison was made by dividing geographic areas into high and low prevalence areas based on the international classification (red areas, ≥40 contaminated per 100,000 habitants and green areas, <40 contaminated per 100,000 habitants over 4 weeks [±2 weeks from the first SMS text]), and classified kidney transplant responders and remaining kidney transplant recipients into residents of red or green areas at the time they answered the questionnaire. A similar analysis was done between kidney transplant responders and the control group.

**Table 2:** Demographic Data for Adult (≥18 y) Norwegian Kidney Transplant Recipients Alive by April 20, 2020, Divided by if They Responded (Kidney Transplant Responders) to the *Life Situation Form* or Not (Remaining Kidney Transplant Recipients)

	Kidney Transplant Responders (n = 1,007)	Remaining Kidney Transplant Recipients (n = 2,589)	*P* Value
Age (y)	56.6 ± 12.6	59.0 ± 15.1	<0.001
Missing data	0	0	
BMI (kg/m^2^)	26.6 ± 5.6	26.6 ± 6.5	>0.99
Missing data	44	124	
Time since transplant (y)	10.2 ± 8.4	10.5 ± 8.4	0.41
Median (25% percentile, 75% percentile)	8.3 (3.6, 14.6)	8.7 (4.0, 14.6)	
Missing data	0	0	
Preemptive transplant	324 (32.2%)	638 (24.6%)	<0.001
Missing data	0	0	
First transplant	838 (83.2%)	2,246 (86.8%)	0.006
Missing data	0	0	
Combined transplantation^a^	51 (5.1%)	152 (5.9%)	0.35
Missing data	0	0	
Living donor	434 (43.1%)	452 (17.5%)	<0.001
Missing data	0	0	
Plasma creatinine (μmol/L)	130 ± 63	133 ± 68	0.23
Missing data	42	120	
Immunosuppression			
Missing data	0	0	
Tacrolimus	635 (63.1%)	1,608 (62.1%)	0.27
Cyclosporine	252 (25.0%)	695 (26.8%)	0.60
Mycophenolate	820 (81.4%)	2,208 (85.3%)	0.01
Prednisolone	941 (93.4%)	2,409 (93.0%)	0.67
mTOR inhibitor	66 (6.6%)	169 (6.5%)	0.98
SARS-CoV-2			
Measured SARS-CoV-2 IgG	815 (80.9%)	1,303 (50.3%)	<0.001
SARS-CoV-2 IgG positive	0 (0.0%)	8 (0.3%)	0.27^b^
COVID-19	0 (0.0%)	20 (0.8%)	0.005
Hypertension, BP ≥140/90 mmHg	423 (43.5%)	887 (35.5%)	0.84
Missing data	36	90	
Taking antihypertensive medication	804 (80.0%)	1,665 (64.6%)	0.19
Missing data	2	12	
BP ≥140/90 mmHg and/or antihypertensive drugs	864 (88.8%)	1,785 (71.3%)	0.26
Missing data	34	85	
Age over 65 y	274 (27.2%)	1,038 (40.1%)	<0.001
Missing data	0	0	

*Note:* Data are presented as mean (± standard deviation) or numbers (%). Data analyzed with R and with independent samples *t* test or χ^2^ test. Abbreviations: BMI, body mass index, BP, blood pressure; COVID-19, coronavirus disease 2019; mTOR; mammalian target of rapamycin; SARS-CoV-2, severe acute respiratory syndrome coronavirus 2.

a Combined with pancreas, liver, or heart.

b Number of positive samples in relation to those taking a sample.

By November 1, 2020, the Norwegian Institute of Public Health reported 1,667,465 polymerase chain reaction tests performed. Among these, 19,563 tests were positive, and the number of deaths was 282 (5.2 per 100,000 inhabitants).^13^ Thus, Norway was categorized as a country with a low infection burden and COVID-19 rate during the first wave.

### Statistical Considerations

Comparison of demographic data between recipients consenting to answer the questionnaire (kidney transplant responders) versus the remaining kidney transplant recipients was performed by the NRR. Data were analyzed using R (version 4.0.4) (Table 1, Table 2) and SPSS (Table 3, Table 4, Table 5), using independent samples *t* test, ^χ2^ test, or Fisher exact test. Results were considered statistically significant when the *P* value was equal to or below 0.05.

**Table 3:** Behavior the Last 2 Weeks Before March 13, 2020

	Kidney Transplant Responders <65 y (n = 725)	Controls <65 y (n = 3,444)	*P* Value	Kidney Transplant Responders ≥65 y (n = 282)	Controls ≥65 y (n = 965)	*P* Value
Not been in a food store	32 (4.4%)	18 (0.5%)	<0.001	28 (9.9%)	22 (2.3%)	<0.001
Missing data	0	3		0	4	
Not been in a queue in a food store	103 (14.2%)	138 (4.0%)	<0.001	67 (23.8%)	117 (12.2%)	<0.001
Missing data	0	3		0	6	
Not been in a mall	119 (16.4%)	237 (6.9%)	<0.001	67 (23.8%)	103 (10.8%)	<0.001
Missing data	0	8		0	10	
Not been closer than 1 m to others in a store	101 (13.9%)	219 (6.4%)	<0.001	74 (26.2)	138 (14.4%)	<0.001
Missing data	0	8		0	8	
Not taken public transport	463 (63.9%)	1,889 (55.0%)	<0.001	203 (72.0%)	564 (58.9%)	<0.001
Missing data	0	7		0	7	
Not taken public transport in rush hours	578 (79.7%)	2,413 (70.3%)	<0.001	260 (92.2%)	798 (83.5%)	0.001
Missing data	0	12		0	9	
No domestic flights	682 (94.1%)	3,038 (88.6%)	<0.01	273 (96.8%)	901 (94.6%)	0.27
Missing data	0	17		0	13	
No international flights	695 (95.9%)	3,107 (90.6%)	<0.01	264 (93.6%)	840 (88.2%)	0.04
Missing data	0	16		0	13	
Not met between 4-9 people	141 (19.4%)	307 (8.9%)	<0.001	109 (38.7%)	194 (20.2%)	<0.001
Missing data	0	5		0	5	
Not met between 10-50 people	332 (45.8%)	966 (28.1%)	<0.001	187 (66.3%)	468 (48.8%)	<0.001
Missing data	0	12		0	6	
Not met more than 50 people	525 (72.8%)	1,991 (58.0%)	<0.001	241 (85.5%)	711 (74.1%)	<0.001
Missing data	0	10		0	6	

*Note:* The kidney transplant responders behaved more carefully than the control group in both age groups (<65 and ≥65 years) in all areas except for domestic flights 2 weeks before March 13. Data analyzed with SPSS and with Fisher exact test. Data are presented as numbers (%).

**Table 4:** Behavior the 2 Weeks After March 13, 2020

	Kidney Transplant Responders <65 y (n = 725)	Controls <65 y (n = 3,444)	*P* Value	Kidney Transplant Responders ≥65 y (n = 282)	Controls ≥65 y (n = 965)	*P* Value
Not been in a food store	94 (13.0%)	51 (1.5%)	<0.001	69 (24.5%)	44 (4.6%)	<0.001
Missing data	0	7		0	5	
Not been in a queue in a food store	267 (36.8%)	437 (12.7%)	<0.001	153 (54.3%)	255 (26.6%)	<0.001
Missing data	0	12		0	6	
Not been in a mall	236 (32.6%)	518 (15.1%)	<0.001	112 (39.7%)	143 (15.0%)	<0.001
Missing data	0	20		0	9	
Not been closer than 1 m to others in the store	341 (47.0%)	964 (28.1%)	<0.001	168 (59.6%)	407 (42.4%)	<0.001
Missing data	0	12		0	6	
Not taken public transport	646 (89.1%)	2,672 (77.8%)	<0.001	260 (92.2%)	794 (83.1%)	<0.001
Missing data	0	9		0	10	
Not taken public transport in rush hours	706 (97.4%)	3,130 (91.2%)	<0.001	282 (100%)	932 (97.4%)	0.02
Missing data	0	11		0	8	
No domestic flights	720 (99.3%)	3,317 (96.5%)	0.03	282 (100%)	943 (98.5%)	0.55
Missing data	0	7		0	8	
No international flights	725 (100%)	3,403 (99.1%)	0.50	279 (98.9%)	945 (98.6%)	>0.99
Missing data	0	11		0	7	
Not met between 4-9 people	437 (60.3%)	1,292 (37.7%)	<0.001	220 (78.0%)	566 (59.0%)	<0.001
Missing data	0	17		0	6	
Not met between 10-50 people	670 (92.4%)	2,787 (81.3%)	<0.001	272 (96.5%)	864 (90.1%)	<0.01
Missing data	0	17		0	6	
Not met more than 50 people	713 (98.3%)	3,307 (96.4%)	0.43	280 (99.3%)	941 (98.3%)	0.14
Missing data	0	15		1	8	

*Note:* The kidney transplant responders and the control group ≥65 years behaved equally carefully with regards to domestic flights. The kidney transplant responders and the control group behaved equally carefully with regards to international flights and avoiding meeting more than 50 people 2 weeks after March 13 in both age groups (<65 and ≥65 years). In all other areas, the kidney transplant responders behaved more carefully than the control group in both age groups (<65 and ≥65 years). Data analyzed with SPSS and with Fisher exact test. Data are presented as numbers (%).

**Table 5:** Behavior the 2 Weeks Before Answering the Questionnaire in the Period Between July 3, 2020, and August 12, 2020

	Kidney Transplant Responders <65 y (n = 725)	Controls <65 y (n = 3,444)	*P* Value	Kidney Transplant Responders ≥65 y (n = 282)	Controls ≥65 y (n = 965)	*P* Value
Not been in a food store	20 (3.6%)	18 (0.7%)	<0.001	13 (5.6%)	13 (1.6%)	<0.01
Missing data	187	950		62	187	
Not taken public transport Missing data	407 (72.9%) 167	1,527 (62.7%) 935	<0.001	181 (77.7%) 49	554 (70.0%) 174	0.03
No international flights	553 (99.1%)	2,474 (98.7%)	0.53	233 (100.0%)	783 (99.1%)	0.36
Missing data	167	937		49	175	
Not met between 4-9 people	133 (24.4%)	324 (12.9%)	<0.001	89 (38.4%)	218 (27.6%)	<0.01
Missing data	170	942		50	175	
Not met between 10-50 people	328 (59.0%)	1,036 (41.3%)	<0.001	176 (76.5%)	479 (60.7%)	<0.001
Missing data	169	935		52	176	
Not met more than 50 people	484 (87.2%)	1,970 (78.6%)	<0.001	215 (92.7%)	680 (86.2%)	<0.01
Missing data	170	939		50	176	

*Note:* The kidney transplant responders and the control group behaved equally careful with regards to international flights in both age groups (<65 and ≥65 years) 2 weeks before answering the questionnaire in the period between July 3, 2020, and August 12, 2020. In all other areas the kidney transplant responders behaved more carefully than the control group in both age groups (<65 and ≥65 years). Data analyzed with SPSS and with Fisher exact test. Data is presented as numbers (%).

### Ethical Considerations

The SMS part of the study was approved by the Norwegian ethics committee (REK 124170) and followed the Helsinki Declaration. It was registered in ClinicalTrials.gov (NTC 04320732). All participants were given information about the study via the digital platform and the right to withdraw from the study at any time. Consent forms were signed electronically. Data collection and storage were administered through the University of Oslo Services for Sensitive Data.

The SARS-CoV-2 IgG screening was separately approved by the Norwegian ethics committee (REK 148904) and followed the Helsinki Declaration.

## Results

Of the 3,060 kidney transplant recipients invited to participate in the SMS study, 1,007 replied to the questionnaire. Of the 20,000 randomly selected individuals from the general Norwegian population, 4,409 answered the same questionnaire and served as a control group.

Demographic characteristics of the kidney transplant responders compared with controls are shown in Table 1. The healthy control group differs from the kidney transplant responders in age (51 vs 57 years; *P* < 0.001), the proportion above 65 years old (22% vs 27%; *P* < 0.001), the proportion of residents in red areas (78% vs 69%; *P* < 0.001) and socioeconomic status (Table 1). As anticipated, there was a substantial difference in the prevalence of hypertension (14% vs 89%; *P* < 0.001) (Table 1).

Demographic characteristics of kidney transplant responders compared with all other kidney transplant recipients are shown in Table 2. The 2 kidney transplant cohorts were evenly distributed within red/green areas and rural and highly populated areas. However, they differed concerning age (57 vs 59 years; *P* < 0.001), the proportion that had undergone preemptive transplant (32% vs 25%; *P* < 0.001), the proportion that had received a kidney from a living donor (43% vs 18%; *P* < 0.001), and the proportion that measured SARS-CoV-2 IgG (81% vs 50%; *P* < 0.001). A significantly higher proportion of those not answering the questionnaire had detectable SARS-CoV-2 Ig antibodies in the screening investigation (0% vs 0.8%). Not surprisingly, the percentage of kidney transplant recipients above 65 years was significantly lower in the kidney transplant responders (Table 2).

The kidney transplant responders were more concerned about being exposed to SARS-CoV-2 infection (27%) than the control group (7%; *P* < 0.001). This was also confirmed in both the age groups ≥65 years and <65 years by the kidney transplant responders’ behavior, as they reported being more careful in their everyday life to limit the risk of SARS-CoV-2 infection than the control group (Table 3, Table 4, Table 5). Both during the 2 weeks before March 13, 2020, and the 2 weeks that directly followed, the kidney transplant responders in both age groups (≥65 years and <65 years) reported behaving more carefully compared with the control group in the respective age group (≥65 years and <65 years) with regards to going to the grocery store, going to the mall, keeping a distance of more than 1 meter from others in the store, and limiting social interactions.

Four of the 1,007 kidney transplant responders stated that they had adjusted their immunosuppressive medications. It is unknown whether this was in conjunction with their nephrologist or on their own initiative.

None of the kidney transplant responders reported a positive nasopharynx test for SARS-CoV-2 polymerase chain reaction in the questionnaire versus 7 participants in the control group (*P* = 0.36). These self-reported numbers have been verified by the Norwegian surveillance system for Communicable Diseases. A total of 815 (81%) of the kidney transplant responders received a SARS-CoV-2 IgG test, and none were positive. There was no significant difference between the groups in the frequency of symptoms compatible with COVID-19 (2.3% in the kidney transplant responders and 2.5% in the control group, *P* = 0.88). Thus, the incidence of COVID-19 and the frequency of symptoms in the 2 groups were not significantly different.

The difference in behavior probably continued throughout the summer as the kidney transplant responders in both age groups (≥65 years and <65 years) reported being more careful in their everyday life than the control group in the respective age group (≥65 years and <65 years) in the period between July 3 and August 18 (Table 5).

## Discussion

We demonstrate that Norwegian kidney transplant study responders adhered closely to nationally initiated COVID-19 preventive measures. None of the kidney transplant responders were infected by SARS-CoV-2 during the first wave of the pandemic. To our knowledge, this is the first study investigating behavioral changes in kidney transplant recipients during the first wave of COVID-19. The study includes answers from a large cohort of kidney transplant recipients and controls and underlines the psychological burden of the COVID-19 pandemic among kidney transplant recipients.

Our results indicate that the kidney transplant responders were more concerned about being infected with SARS-CoV-2 than the general population. This finding was anticipated, as kidney transplant recipients are immunosuppressed and have more comorbid conditions than the general population. On March 12, 2020, the Norwegian government introduced the strongest and most intrusive measures during peacetime in Norwegian history to prevent increasing infection rates. The Norwegian population followed the government’s general advice on infection control to a large degree. Being informed on the risk of developing severe disease if infected with SARS-CoV-2, our data suggest that Norwegian kidney transplant recipients behaved even more carefully than the general Norwegian population during the first wave of COVID-19 in Norway. It is often stated that persons with a high socioeconomic status behave more appropriately in difficult settings than persons with a lower socioeconomic status. In our study, the kidney transplant recipients had a slightly lower socioeconomic status (income, education) than the control group. Despite this, the kidney transplant responders behaved more carefully compared with the control group. Compared with countries with a high^7^, ^8^, ^9^ prevalence of COVID-19, our data from the first wave indicate that Norwegian kidney transplant recipients have a similar prevalence of COVID-19 as the general population. This finding seems persistent throughout waves 2 and 3 as we, up to March 2021, have 47 SARS-CoV-2-positive (by polymerase chain reaction) kidney transplant recipients in Norway, representing a prevalence of 1.3% (47/3,596). This is similar to the prevalence in the total Norwegian population, which is 1.4% (73,000/5.4 million). These findings are somewhat unexpected as this patient group has a high number of comorbid conditions, uses immunosuppressive medications, and has frequent contact with the health care system. The explanation lies in the fact that our kidney transplant recipients have behaved more carefully and followed governmental advice during the first wave of COVID-19. The low incidence of COVID-19 in the Norwegian population, which most likely reduced the infection risk among kidney transplant recipients, is consistent with Australian findings.^14^ Contrary to this, a study from France, a high-prevalence COVID-19 country, reported a higher prevalence among kidney transplant recipients (5%) compared with the general population (0.3%).^7^ In this report, kidney transplant recipients’ mortality was substantially higher than among the general population (24% vs 1%). In Norway, preliminary data indicate mortality at approximately 20% among kidney transplant recipients infected with SARS-CoV-2 during the first wave.^10^ Throughout the second and third waves, the mortality rate in Norway seemed somewhat reduced, with 7 deceased among 47 infected (14%) up to March 2021. It is known that exposure to a high viral load during contamination is correlated with more serious COVID-19 disease.^15^, ^16^, ^17^, ^18^ Thus, one can suggest that cautious behavior is associated with less risk of viral transmission and consequently milder disease. One could also speculate that the population pattern in Norway, with people living in remote areas and small cities yielding a low population density (15 persons/km^2^ compared with 120 persons/km^2^ in France), makes spreading of SARS-CoV-2 more unlikely and thus represents a “protective feature” of the country.

During a pandemic, there are frequent distributions of misleading information, specifically on social media. As kidney transplant responders were more concerned about infection with SARS-CoV-2 and simultaneously knew that immunosuppression was a “risk factor,” one could assume that some would react to symptoms or suspicion of COVID-19 by reducing their dose of immunosuppressive medication as a precautionary measure. This would lead to an increased risk of graft rejection. However, only 4 of the 1,007 kidney transplant responders reported having changed their immunosuppressive medications during the investigation period. This probably corresponds with the low incidence of life-threatening COVID-19 disease among Norwegian kidney transplant recipients. Valid COVID-19 information was posted and regularly updated on the national association’s homepage for kidney patients and transplant recipients. Kidney transplant recipients were informed not to reduce immunosuppressive drugs on their own but only in case of serious disease, and then in conjunction with the treating transplant physician. This indicates that kidney transplant recipients have received good and safe follow-ups in the health care system and that the patients have been adherent. Therefore, we do not expect an increased risk of (chronic or antibody-mediated) transplant rejection among Norwegian kidney transplant recipients following the first wave of COVID-19.

The present study has some limitations. First, the number of SARS-CoV-2-infected kidney transplant recipients in Norway is low, and mortality data is therefore not reliable. However, the second and third waves’ mortality is still 14%, lower but comparable to reports from other European countries.^10^ Second, the relatively low questionnaire response rate for kidney transplant recipients is not optimal. On the other hand, the comparison between the kidney transplant responders and all other kidney transplant recipients suggests that those answering the questionnaire are representative of the whole group regarding the risk of adverse COVID-19 outcomes. Third, it may be possible that there is a selection bias, as the kidney transplant responders might be more careful than the rest of the kidney transplant population. However, this would probably also be the case for the control group.

Thus, our findings suggest that even though kidney transplant recipients might be at increased risk of SARS-CoV-2 infection and critical COVID-19 illness, Norwegian kidney transplant recipients as a group have been relatively “safe” during the first wave of COVID-19. The more careful social behavior of the recipients, and the low incidence of COVID-19 in the general population, are probably important contributors to this finding. Hopefully, continuous watchful behavior will prevent increased mortality among kidney transplant recipients until the vaccine ends the pandemic.
